# A comparison of pathways to care in at-risk mental states and first episode psychosis: a mental health electronic clinical records analysis in the East of England, UK

**DOI:** 10.1007/s00127-025-02833-3

**Published:** 2025-03-01

**Authors:** Rhiannon Murden, Sophie M. Allan, Jo Hodgekins, Sheri Oduola

**Affiliations:** 1 Norwich Medical School, University of East Anglia, Norwich Research Park, Norwich, NR4 7TJ UK; 2 Cambridgeshire and Peterborough NHS Foundation Trust, Elizabeth House, Fulbourn, Cambridgeshire, CB21 5EF UK; 3 School of Health Sciences, University of East Anglia, Norwich Research Park, Norwich, NR4 7TJ UK; 4 Birmingham and Solihull Mental Health NHS Foundation Trust, Uffculme Centre, 52 Queensbridge Road, Moseley, Birmingham, B13 8QY UK

**Keywords:** At risk mental states, First episode psychosis, Early intervention, Pathways to care, Sociodemographic determinants

## Abstract

**Purpose:**

Delays in treatment for individuals experiencing early signs of psychosis are associated with poorer outcomes. Few people presenting with first episode psychosis (FEP) access early intervention in psychosis (EIP) services during the prodromal stage. In this study, we compared pathways to care (PtC) in people with At-Risk Mental States (ARMS) and FEP and explored the sociodemographic factors associated with accessing EIP during ARMS or FEP.

**Methods:**

Sociodemographic and PtC data were collected from the Cambridgeshire and Peterborough NHS Foundation Trust (CPFT) Research Database. All individuals referred and accepted to CPFT EIP services as either ARMS or FEP between 1st April 2018 and 31st October 2019 (N = 158) were included.

**Results:**

There was strong evidence that ARMS patients accessing EIP were younger and were less likely to have a minority ethnic status than FEP patients. In terms of PtC, ARMS patients had fewer numbers of contacts, were less likely to be referred via the acute services, less likely to be involuntarily admitted and had reduced family involvement in their help-seeking. No differences were identified between ARMS and FEP in terms of living circumstances, area-level deprivation, urbanicity, employment status, duration of PtC, or police involvement in PtC.

**Conclusion:**

Our findings highlight that disparities exist between ARMS and FEP patients in terms of sociodemographic and PtC characteristics. Further research is required to replicate these findings and investigate the effectiveness of interventions to encourage and facilitate access to EIP at an earlier stage to improve outcomes.

## Introduction

The earlier people receive appropriate treatment for first episode psychosis (FEP) the better their outcomes [1]. Longer duration of untreated psychosis (DUP) is associated with lower overall functioning, more severe symptoms, lower quality of life, and reduced likelihood of full remission [2–4]. Additionally, longer DUP is associated with increases in both direct and indirect economic costs [5] and ultimately results in prolonged distress for the individual and their families [6].

Interventions could occur at an even earlier stage of illness, when individuals are at high risk of developing psychosis, referred to as “At-Risk Mental State” (ARMS) [7]. ARMS is characterised by psychotic symptoms of lesser severity and duration than FEP and accompanied by a drop in functioning [7]. It has been suggested that receiving interventions during the ARMS period may alter outcome trajectories by reducing DUP, therefore improving outcomes, or preventing transition to psychosis altogether [7, 8]. However, only a small proportion of individuals presenting with FEP have been identified by prodromal services [9].

Given how few people presenting with FEP reach prodromal services [9], it is important to investigate pathways to care (PtC): the time between help-seeking initiation, and receiving appropriate intervention [10], for individuals who present during earlier stages of illness (ARMS) and those who do not (FEP). It is crucial that PtC for individuals with ARMS and FEP are as direct, timely, and straightforward as possible owing to the importance of achieving better recovery outcomes through a shorter DUP [1, 3, 6]. Despite this, PtC for people with psychosis are often complex and involve multiple contacts with different services [11] and lengthy delays [6].

In FEP, several social and demographic factors have been found to be associated with longer and more negative PtC. Negative PtC have been defined as contacts with police and emergency services, crisis teams, and compulsory inpatient admissions [12–14]. Living alone [15], lack of family involvement [16], unemployment [16, 17], being a first-generation immigrant [18], or from an ethnic minority background [16], living in a rural area [18, 19], or areas with higher-than-average social deprivation [20], and being male [21, 22] have all been associated with longer and more negative PtC.

Literature regarding PtC for ARMS is scarcer than FEP. A recent systematic review [23] found that only a small percentage of ARMS patients had PtC via emergency services or compulsory admissions, with first help-seeking contacts more commonly made through a GP or mental health professional. The review found some evidence that family involvement may support help-seeking in ARMS [23].

Current evidence indicates differences in sociodemographic and PtC characteristics between ARMS and FEP populations. Few people with ARMS experience compulsory admission or emergency service involvement in their PtC [23], whereas this is frequently observed in the PtC of individuals with FEP [11]. Although limited, current evidence suggests that rates of ARMS identification and transition to FEP for those with ethnic minority and migrant status is inconsistent with the elevated incidence of psychotic disorders in these populations [24–26]. The authors suggest this may be because migrants or individuals from ethic minority groups do not reach services during ARMS [24]. Additionally, research suggests a higher incidence of women identified during ARMS [27] compared to higher incidence of men identified during FEP [28].

Whilst differences can be expected between ARMS and FEP populations, there is limited research directly comparing sociodemographic and PtC characteristics for ARMS and FEP. This is important as it would further our understanding of factors associated with individuals who present during earlier stages of illness (ARMS) and those who present with FEP. To date there are four studies directly comparing the groups. Two were conducted in Switzerland and found no significant differences between ARMS and FEP on duration or number of PtC [29, 30]. One study was conducted in the United States and found no differences in duration of PtC or sociodemographic characteristics between ARMS and FEP [14]. It is worth noting that the healthcare systems in these countries differ to the United Kingdom (UK) National Health Service (NHS) and therefore PtC also likely differ. The final study, was a qualitative study carried out in a UK, NHS setting which identified common themes between experiences of PtC for both groups including negative experiences of PtC and significant treatment delays [31].

To our knowledge there have been no quantitative studies conducted in the UK, comparing PtC for individuals with ARMS and FEP. Therefore, in this study, we sought to compare PtC in ARMS and FEP in UK Early Intervention in Psychosis (EIP) services and explore factors which may be associated with accessing treatment at an earlier stage of illness. We addressed the following research questions: 1) Are there differences in PtC characteristics between individuals with ARMS and FEP? 2) Do individuals with ARMS and FEP differ by sociodemographic characteristics? 3) Are any of the sociodemographic and PtC factors predictive of whether someone seeks help during ARMS compared with FEP, independent of confounders?

## Methods

### Design

A cross-sectional design was employed.

### Study setting and participants

The study was conducted within the area of Cambridgeshire and Peterborough in the East of England, UK. According to the 2021 census [32], in both Cambridgeshire and Peterborough the largest proportion of people are aged between 35–49 years (Cambridgeshire: 19.8%, Peterborough: 21.5%), are female (Cambridgeshire and Peterborough: 50.6%), identify as White British or White Non-British (Cambridgeshire: 88.6%, Peterborough: 75.4%) and are Employed (Cambridgeshire: 58.2%, Peterborough: 58.9%).

Participants were identified from Cambridgeshire and Peterborough NHS Foundation Trust (CPFT) EIP services, providing assessment and intervention to people presenting with ARMS (as assessed by the Comprehensive Assessment of At-Risk Mental States) [33] or FEP. CPFT serves a population of approximately 950,000 across Cambridgeshire and Peterborough including both rural and urban areas [34], and affluent and deprived areas [35]. Referrals to CPFT EIP services are accepted from any source.

### Procedure

Data were collected from all individuals accepted onto the EIP caseload as either ARMS or FEP between 1st April 2018 and 31st October 2019, using the CPFT Research Database (CPFTRD) [36]. This period was selected to include the most recent cohort of EIP users prior to the COVID-19 pandemic. It was anticipated that access and use of services would have been adversely impacted by the pandemic [37], however this was not the focus of the present study.

We used the Clinical Records Anonymisation and Text Extraction (CRATE) [38] in CPFTRD for data extraction. CPFTRD is a database of de-identified clinical records used for research purposes and contains information from approximately 260,000 people receiving care from CPFT [39]. The research database contains structured data fields (including demographic variables and dates) and unstructured free-text fields (including clinical information from clinical documents, assessments, and progress notes). The database contains information pertaining to care received from secondary mental health, psychiatric liaison, and psychiatric inpatient services within CPFT and sources of referral.

### Case identification, inclusion/exclusion criteria

Initial searches of the database were conducted to identify individuals referred and accepted onto the EIP caseload between 1st April 2018 and 31st October 2019. Each individual record was then screened manually to determine if the individual met the study eligibility criteria. We included individuals if they were:

- presenting with and clinically assessed as ARMS or FEP
- accepted to a CPFT EIP caseload during the study period
- residing in the Cambridgeshire and Peterborough catchment areas during the study period
- aged 14- to 35-years old

We excluded individuals who were deemed by clinicians not to be presenting with psychosis and were not accepted to a CPFT EIP service.

Following the introduction of the NHS Access and Wait Time Standards for EIP [40] CPFT EIPs extended the age acceptability criterion for FEP from 14–35 years to 14–65 years [41] but not for ARMS. Therefore, we restricted our analyses to those aged 14–35 years in both ARMS and FEP groups.

### Data collection and instruments

#### Sociodemographic characteristics

Sociodemographic data were collected using an adapted version of the Medical Research Council Sociodemographic Schedule (MRC-SDS) [42]. This measure has been widely used in previous studies to collect sociodemographic characteristics [43–46]. Sociodemographic information collected included age at EIP assessment, gender, ethnicity, living circumstances and employment status.

**Ethnicity** was classified according to the 18 categories used by the UK Office of National Statistics (ONS) [47] within the CPFTRD. Due to the small number of patients belonging to minority ethnic groups and for data analysis purposes, we collapsed ethnicity into four categories: White British; White non-British (white Irish, white Gypsy, white Other); Any Other Ethnic Groups (black African, black Caribbean, other black, any mixed ethnic group, Indian, Pakistani, Bangladeshi, Chinese, other Asian, any other ethnic group); Not Stated. This is consistent with previous studies [45, 48].

**Living circumstances** were coded as binary: living alone or living with others (i.e., with family/friends, in supported or sheltered accommodation, and within student accommodation).

**Employment status** was categorised as Employed; Unemployed; Student. The ONS statistical bulletin [49] categorised individuals as either employed or unemployed, it highlighted higher rates of unemployment in younger people and suggested this may be linked to staying in education for longer. The EIP accepts individuals from the age of 14, and therefore will see several younger people who may be in full-time education. As a result, the additional category of “student” was included consistent with previous studies [48, 50].

Additional socio-environmental information pertaining to ARMS and FEP patients’ rural/urban and area-level deprivation status was collected. In CPFTRD, patients’ residential addresses e.g., postcodes are replaced with administrative geographical level of Lower Super Output Area (LSOA) information.

**Rural–urban status** was determined using the ONS Rural–Urban Classifications linked to LSOA [51]. The ONS Rural–Urban Classification assigns areas to one of four urban categories (major conurbation; minor conurbation; city and town; city or town in sparse settings) or six rural categories (town or fringe; town or fringe in sparse settings; village; village in sparse settings; hamlets and isolated dwellings; hamlets and isolated dwellings in a sparse setting) [51]. These categories were collapsed into two: urban and rural, in line with the ONS guidelines [51].

**Area-level deprivation** is linked to de-identified clinical records in CPFTRD using the index of multiple deprivation (IMD) score, which is a measure of relative deprivation for small areas of England, ranking areas from one (most deprived) to 32,844 (least deprived) [52]. These ranks were collapsed into quintiles from one (most deprived) to five (least deprived).

#### Pathways to care

PtC data were extracted from CPFTRD for each individual using an adapted version of the Personal and Psychiatric History Schedule [53] consistent with previous research investigating PtC [44, 45, 54].

**Duration of PtC** was measured from the date of referral into CPFT services (leading to EIP referral), to the date of EIP assessment.

**Number of PtC** was defined as the number of referrals accepted to CPFT services (including EIP) during this time.

**Mode of contact** was classified as the source of referral to EIP and categorised as Primary Services (e.g., GP and primary mental health services); Secondary Services (e.g., community mental health teams); Acute Services (e.g., accident and emergency, crisis, and inpatient services); Informal (e.g., self, family, or non-mental health organisations such as educators or charities).

**Additional PtC data** were collected pertaining to whether an individual had been detained under the Mental Health Act (MHA); a legal framework allowing for involuntary hospital admission for mental health problems [55]. We also collected data on family/friends, or police or criminal justice services involvement, in PtC during the period between first CPFT contact and EIP assessment. Involvement of family/friends was classified as family/friends initiating or supporting help-seeking and included initiating referrals or contact with services for advice or supporting patients to appointments. Police or criminal justice involvement was classified as contact with police or criminal justice system for reasons relating to presenting difficulties resulting in EIP assessment. For example, being detained under Section 136 of the MHA (in which police hold power to remove individuals who appear to require immediate mental health care from public places to places of safety) [55], arrests or criminal proceedings, or telephone calls to the police with concerns about the individual’s behaviour.

### Reliability

Steps were taken to ensure the reliability of data collection procedures from the de-identified clinical records. Each variable was operationalised and a document was produced indicating where information could be found in CPFTRD. This was used by RM for data collection and SO for data checking. Data on around fifteen percent (*n* = 21) of the sample were checked by SO who was blind to the original extraction. A kappa score of 0.81 (*p* < 0.001), and 90.5% agreement was achieved for ARMS or FEP information. A kappa score of 0.65 (*p* < 0.001), and 71.4% agreement was achieved for number of PtC.

### Ethical approval

The CPFTRD was approved by an NHS Research Ethics Committee (reference: 17/EE/0442) for secondary analysis. This study was also granted ethical approval by the London-Chelsea Research Ethics Committee (reference: 19/LO/0398). Local approval was obtained from the CPFTRD Oversight Committee (reference: M00921). Under UK law, participant consent was not required for this study [36].

### Statistical analysis

Data were analysed using PSPP [56]. An alpha level of *p* = 0.05 was used for all analyses. Descriptive statistics including frequencies and percentages for categorical variables, mean and standard deviation, or median and ranges for continuous variables were used to describe the sample.

Continuous data were checked for normality. Independent t-tests were used for normally distributed data and Mann–Whitney U tests were used for non-parametric continuous variables. We performed chi-square or Fisher’s exact for categorical variables to determine if there were significant differences between participants with ARMS and FEP, based on sociodemographic and PtC variables. Variables demonstrating statistically significant differences between individuals with ARMS and FEP were first tested individually with univariate binary logistic regression (Model 1) followed by controlling for *a-priori* confounders (age, gender, and ethnicity) in Model 2. These provided estimates of crude and adjusted odds ratios of associations between PtC and sociodemographic characteristics among ARMS and FEP patients.

## Results

### Sample selection

**Fig. 1:**
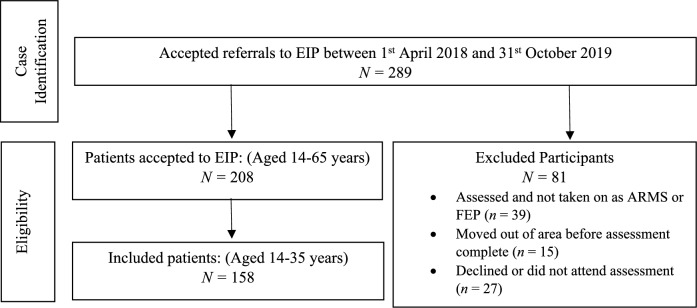
Flow diagram of the inclusion process

A flow diagram of case identification is given in Fig. 1. Initial searches of the CPFTRD returned 289 patients referred to EIP between 1st April 2018 and 31st October 2019. Of these, 208 patients aged 14–65 years were accepted to the service as either ARMS or FEP, of whom 158 were aged 14–35 years. Data and results presented here are on the 158 patients aged 14–35 years accessing EIP as ARMS or FEP.

### Sample characteristics

Sociodemographic and PtC descriptive statistics are shown in Table 1. The mean age of was 23.95 (SD = 5.4) and there were more men (61.4%) than women (38.6%). Most patients were White British (62%), from urban areas (73.4%), and living with others (88.6%). There was a similar proportion of patients in terms of employment status. The largest proportion of patients were within the least deprived quintile (28.5%). More patients were accepted by EIP as FEP (57.6%) compared to ARMS (42.4%).

**Table 1:** Sample characteristics

	N = 158 (%)
Diagnosis	
ARMS	67 (42.4)
FEP	91 (57.6)
Mean age (sd) years	23.95 (5.4)
Gender	
Men	97 (61.4)
Women	61 (38.6)
Ethnicity	
White British	98 (62.0)
White Non-British	19 (12.0)
Any Other Ethnic Group	34 (21.5)
Not Stated	7 (4.4)
Living Circumstances	
Alone	11 (7.0)
With Others	140 (88.6)
Missing	7 (4.4)
Mean IMD Scores (sd)	17,650.03 (9942.6)
IMD Quintiles	
1 (Most Deprived)	24 (15.2)
2	29 (18.4)
3	32 (20.3)
4	25 (15.8)
5 (Least Deprived)	45 (28.5)
Missing	3 (1.9)
Urbanicity	
Rural	39 (24.7)
Urban	116 (73.4)
Missing	3 (1.9)
Employment Status	
Employed	58 (36.7)
Student	49 (31.0)
Unemployed	51 (32.3)
Median Duration of PtC (range) days	12 (0–312)
Median Number of PtC (range)	2 (1–28)
Mode of Contact	
Primary Services	47 (29.7)
Secondary Services	29 (18.4)
Acute Services	61 (38.6)
Informal	21 (13.3)
Detained under MHA	
Yes	28 (17.7)
No	130 (82.3)
Family/Friend Involvement	
Yes	103 (65.2)
No	55 (34.8)
Police Involvement	
Yes	34 (21.5)
No	124 (78.5)

*SD* Standard Deviation, *PtC* Pathways to Care, *MHA* Mental Health Act, *IMD* Index of Multiple Deprivation

The median duration of PtC was 12 days (range = 0–312) and the median number of PtC contacts was 2 (range = 1–28). During their PtC, there were fewer patients detained under the MHA (17.7%) and less police or criminal justice involvement (21.5%). Family/friend involvement in help-seeking was common (65.2%). Mode of contact with EIP varied, with most patients being referred by acute services (38.6%) followed by primary services (29.7%).

### Comparisons between ARMS and FEP by sociodemographic and pathways to care characteristics

Descriptive comparisons of PtC and sociodemographic variables between ARMS and FEP patients are summarised in Table 2. The number of PtC contacts were fewer for ARMS patients (median = 1, range = 1–9) compared to FEP patients (median = 3, range = 1–28) (Mann–Whitney *U*: 1905.5, *p* < 0.001). Compared with FEP patients, ARMS patients were more likely to contact EIP via primary care (ARMS:43.3%, FEP:19.8%) and less likely to be referred via acute services (ARMS:17.9%, FEP:53.8%) (Χ^2^ = 23.41, *df* = 3, *p* < 0.001). Conversely, FEP patients were more likely to be admitted involuntarily (ARMS:3%, FEP:28.6%) (Χ^2^ = 17.33, *df* = 1, *p* < 0.001) and more likely to have family/friend involvement (ARMS:49.3%, FEP:76.9%) (Χ^2^ = 13.02, *df* = 1, *p* < 0.001) than ARMS patients. We found no differences in police and/or criminal justice involvement or duration of PtC between ARMS and FEP patients.

**Table 2:** Comparison between ARMS and FEP by sociodemographic and pathways to care characteristics

	ARMS *n* = 67(%)	FEP *n* = 91(%)	Χ^2^/t-/Mann Whitney tests, (df), *p*
Mean age (sd) years	22.26 (5.03)	25.19 (5.32)	*t* = −3.50, *p* = 0*.*001**
Gender			
Men	36 (53.7)	61 (67.0)	2.88 (1), *p* = 0.09
Women	31 (46.3)	30 (33.0)	
Ethnicity			
White British	53 (79.1)	45 (49.5)	15.94 (3), *p* = 0.001**
White Non-British	3 (4.5)	16 (17.6)	
Any Other Ethnic Group	8 (11.9)	26 (28.6)	
Not Stated	3 (4.5)	4 (4.4)	
Living Circumstances†			
Alone	5 (7.9)	6 (6.8)	0.07 (1), *p* = 0.794
With Others	58 (92.1)	82 (93.2)	
Mean IMD Scores (sd)	18,378.42 (9324.5)	17,113.75 (10,392.4)	*t* = 0.79, *p* = 0.431
IMD Quintiles†			
1 (Most Deprived)	7 (10.4)	17 (19.3)	3.74 (4), *p* = 0.442
2	16 (23.9)	13 (14.8)	
3	13 (19.4)	19 (21.6)	
4	11 (16.4)	14 (15.9)	
5 (Least Deprived)	20 (29.9)	25 (28.4)	
Urbanicity†			
Rural	20 (29.9)	19 (21.6)	1.38 (1), *p* = 0.24
Urban	47 (70.1)	69 (78.4)	
Employment Status			
Employed	25 (37.3)	33 (36.3)	0.05 (2), *p* = 0.977
Student	21 (31.3)	28 (30.8)	
Unemployed	21 (31.3)	30 (33.0)	
Median Duration of PtC (range) days	14 (0–182)	12 (0–312)	*U*: 2940, *p* = 0.702
Median Number of PtC (range)	1 (1–9)	3 (1–28)	*U*: 1905.5, *p* < 0.001**
Mode of Contact			
Primary Services	29 (43.3)	18 (19.8)	23.41 (3), *p* < 0.001**
Secondary Services	13 (19.4)	16 (17.6)	
Acute Services	12 (17.9)	49 (53.8)	
Informal	13 (19.4)	8 (8.8)	
Detained under MHA			
Yes	2 (3.0)	26 (28.6)	17.33 (1), *p* < 0.001**
No	65 (97.0)	65 (71.4)	
Family/Friend Involvement			
Yes	33 (49.3)	70 (76.9)	13.02 (1), *p* < 0.001**
No	34 (50.7)	21 (23.1)	
Police Involvement			
Yes	11 (16.4)	23 (25.3)	1.79 (1), *p* = 0.181
No	56 (83.6)	68 (74.7)	

*SD* Standard Deviation, *PtC* Pathways to Care, *MHA* Mental Health Act, *IMD* Index of Multiple Deprivation

^†^ Incongruent n is due to missing data

^*^p ≤ .05

^**^p ≤ .001

In terms of sociodemographic characteristics, compared with FEP patients, ARMS patients were significantly younger (ARMS: Mean = 22.26, SD = 5.03, FEP: Mean = 25.19, SD = 5.32) (*t* = -3.5,* p* = 0.001)., and more likely to be White British (ARMS: 79.1%, FEP: 49.5%) (Χ^2^ = 15.94, *df* = 3, *p* = 0.001). No differences were observed between ARMS and FEP patients by gender, living circumstances, IMD scores, rural/urban, or employment status.

### Crude and multivariable logistic regression analysis of associations with pathways to care and sociodemographic characteristics in ARMS and FEP

In the unadjusted binary logistic regression model, there was strong evidence of an association between age, ethnicity, number of PtC, mode of contact, detention under the MHA, and having family/friend involvement in PtC and accessing EIP during ARMS compared to FEP (Model 1, Table 3). After adjusting for *a-priori* confounding variables (age, gender, and ethnicity) there remained strong evidence of an association between PtC and sociodemographic variables and accessing EIP during ARMS compared to FEP (Model 2, Table 3). Compared with FEP patients, ARMS patients were younger (adjusted OR = 0.89, CI = 0.82–0.95), less likely to be White Non-British (adjusted OR = 0.17, CI = 0.04–0.66) and Any Other Ethnicity (adjusted OR = 0.18, CI = 0.07–0.48). There was strong evidence that ARMS patients were less likely to access EIP via acute services (adjusted OR = 0.17, CI = 0.06–0.45) and less likely to be detained under the MHA (adjusted OR = 0.10, CI = 0.02–0.45). We found strong evidence ARMS patients were less likely to have family/friend involvement (adjusted OR = 0.33, CI = 0.15–0.72) in their access to EIP. We found weak evidence that ARMS patients were less likely to be male (adjusted OR = 0.50, CI = 0.24–1.05), and less likely to access EIP via secondary services (adjusted OR = 0.37, CI = 0.13–1.07).

**Table 3:** Crude and Multivariable Logistic Regression Analysis of Associations with Pathways to Care and Sociodemographic Characteristics in ARMS and FEP

	Model 1		Model 2	
	Unadjusted OR	95% CI	Adjusted OR	95% CI
Mean age (sd) years	0.89	0.84–0.96**	0.89	0.82–0.95**
Gender				
Female	1.00		1.00	
Male	0.57	0.30–1.09	0.50	0.24–1.05
Ethnicity				
White British	1.00		1.00	
White Non-British	0.16	0.04–0.58*	0.17	0.04–0.66*
Any Other Ethnic Group	0.26	0.11–0.63*	0.18	0.07–0.48**
Not Stated	0.64	0.14–3.00	0.91	0.16–5.07
Number of PtC	0.67	0.55–0.82**	0.72	0.59–0.87**
Mode of Contact				
Primary Services	1.00		1.00	
Secondary Services	0.50	0.20–1.29	0.37	0.13–1.07
Acute Services	0.15	0.06–0.36**	0.17	0.06–0.45**
Informal	1.01	0.35–2.91	1.13	0.33–3.86
Detained under MHA				
No	1.00		1.00	
Yes	0.08	0.02–0.34**	0.10	0.02–0.45*
Family/Friend Involvement				
No	1.00		1.00	
Yes	0.29	0.15–0.58**	0.33	0.15–0.72*

Model 1—Unadjusted

Model 2—Adjusted for age, gender, ethnicity

*OR* Odds Ratio, *CI* Confidence Interval, *SD* Standard Deviation, *PtC* Pathways to Care, *MHA* Mental Health Act

^*^p ≤ .05

^**^p ≤ .001

## Discussion

### Main findings

This study compared PtC and sociodemographic characteristics for individuals with ARMS and FEP accessing EIP services in Cambridgeshire and Peterborough, UK. It explored whether any of these characteristics were predictive of accessing EIP during ARMS compared to FEP. There was strong evidence ARMS patients were younger, less likely to have a minority ethnic background, have fewer PtC contacts, and less likely to access EIP via acute services, compared to FEP patients. In addition, ARMS patients were less likely to be involuntarily admitted or have family/friend involvement during their PtC. There was weak evidence to suggest ARMS patients were less likely to be men. We found no differences between ARMS and FEP in terms of living circumstances, deprivation, urbanicity, employment status, duration of PtC, or police involvement.

### Interpretation of findings

#### Pathways to care

The definition of PtC used for this study was the time between the individual’s first referral into CPFT services and EIP assessment, based on data available in the CPFTRD. Apart from the referral source into CPFT (such as primary care or self-referrals), it was not possible to collect data on length of help-seeking and contacts outside of secondary mental health services. This is an important consideration when interpreting the study findings.

In contrast with previous research which found no difference in the number of PtC contacts between ARMS and FEP [29, 30], we found ARMS patients were more likely to have fewer PtC contacts than FEP patients. Whilst we found no significant differences in duration of PtC between ARMS and FEP patients, consistent with previous research [14, 29, 30], the median duration of PtC within CPFT was short for both groups (14 and 12 days for ARMS and FEP patients respectively) and significantly shorter than reported in previous research [14, 29–31]. The relatively short delays observed once referred to secondary mental health services may be accounted for by the introduction of the Access and Waiting Time Standards in 2016 which highlighted the need for, and duty of secondary services to rapidly refer those suspected of experiencing FEP to EIP services [57].

These contrasting results likely reflect differences in PtC definition and suggest PtC prior to accessing secondary mental health services may be longer than within such services [58] and ARMS patients may have more PtC contacts outside of secondary mental health services [29–31]. Given this, it may be that PtC prior to reaching secondary care services constitute the most delay to treatment. Therefore, future research should try to understand these PtC for both ARMS and FEP populations in order to inform the development of targeted interventions to promote earlier engagement in treatment.

Our finding that ARMS patients were more likely than FEP patients to access EIP via primary care services than acute services, or to a lesser extent, secondary services, chimes with previous research. For example, contact with emergency services and inpatient admissions are common in FEP [11] and poor awareness or insight into illness may impede help-seeking [59] resulting in more contacts within secondary and acute mental health services. In keeping with previous research, ARMS patients were less likely to be detained under the MHA during their PtC than FEP patients [31, 60, 61]. No differences were found between ARMS and FEP in terms of police or criminal justice involvement which is consistent with previous findings [14]. This is surprising given that systematic reviews have identified that police and emergency service contact accounts for a small proportion of PtC in ARMS [23] and are relatively frequent in FEP [11]. It may be that this finding is a reflection of the awareness of EIP services within UK secondary mental health service [57], therefore individuals that might otherwise have had criminal justice involvement may have been referred to EIP directly. It is also possible that individuals experienced more criminal justice involvement which did not result in a referral to secondary mental health services and therefore were not captured in this study.

Although family/friend involvement during PtC were common for both groups, our data showed ARMS patients were less likely than FEP patients to have family/friend involvement [14, 29]. We were surprised by this finding. A possible explanation could be limited insight, as FEP patients more often rely on others to seek-help on their behalf [14, 31]. Additionally, early, non-specific symptoms experienced by individuals with ARMS [7, 33] may be less easily detected by others than positive psychotic symptoms [23, 62]. Alternatively, given the high proportion of students in the sample (31%) it is possible that individuals may have been supported by educators rather than family/friends during their PtC. Indeed, educators often play an important role in the early detection of young people’s mental health [63, 64]. It was, however, not possible to capture information pertaining to help-seeking contacts within non-healthcare settings such as the education sector in this study.

#### Sociodemographic factors

One previous study from the United States directly compared sociodemographic characteristics between ARMS and FEP and found no differences in gender, ethnicity, accommodation or household income [14]. In contrast, our study found that being younger and White British were strongly associated with accessing EIP during ARMS compared to FEP. This is unsurprising, given that being from an ethnic minority background has been associated with prolonged PtC within the FEP literature [16]. Evidence suggests that treatment delays are significantly longer for first generation immigrants [18] and patients of Black ethnicity are more likely to have longer PtC [13]. To date within the ARMS literature, the effect of ethnicity on PtC has been neglected [23].

Further research is warranted with patients from diverse backgrounds, this will provide a more nuanced understanding of the influence of ethnicity on help-seeking for ARMS and help develop culturally appropriate strategies to facilitate timely access to care [13].

Our findings indicated a weaker association between gender and accessing EIP during ARMS, with women more likely to access EIP during ARMS compared to men. A larger sample size may have found stronger evidence for this association. Although Fridgen and colleagues [29] did not find a significant difference between ARMS and FEP patients in terms of gender, they found differences in the help-seeking patterns of men and women. Women seemed more likely to seek help from mental health professionals than men [29]. Evidence suggests that women may have more positive attitudes towards seeking psychological help [65], whereas men described difficulties in talking about symptoms and believed help-seeking was perceived as weakness by their peers [66]. These beliefs may mean men do not seek support during earlier stages of illness, resulting in the need for more crisis interventions such as involuntary admissions. An important limitation of this study is that gender is recorded in a binary way within the CPFTRD, and therefore gender-diversity is not accounted for in the findings. Future research is warranted to investigate whether this would impact on accessing EIP services at an earlier stage given there is evidence to suggest gender-diverse individuals may face barriers to accessing mental health services [67].

Evidence within FEP research suggests living alone [15] being unemployed [16, 17], socioeconomic deprivation [68] and rural living [18, 19] are associated with longer PtC. Our findings suggest these factors are not significantly associated with accessing EIP during ARMS compared to FEP.

### Strengths and limitations

To our knowledge, this study was the first quantitative study conducted in a UK, NHS setting exploring sociodemographic and PtC variables associated with accessing EIP during ARMS compared to FEP. We included all individuals accepted onto EIP services of a large mental health provider. Our case identification and data extraction procedures were based on previous electronic health records [44] hence providing a robust and representative sample of ARMS and FEP patients presenting to services during the study period.

Limitations should be considered. Firstly, due to the availability of information on the CPFTRD, duration, and number of PtC information was limited to those which occurred within CPFT and provides an estimate of treatment delays within secondary mental health services. Therefore, a comprehensive PtC: time between onset, help-seeking, and receiving appropriate treatment has not been achieved. This could have been improved through data linkage e.g. to primary care research databases. Additionally, since our sample was drawn from EIP services only and restricted to individuals aged 14–35 years, it is inevitable that patients presenting to other services in CPFT were excluded. Consequently, our findings may not chime with other studies that extend their case identification beyond EIP services or those aged over 35-years old.

The relatively small sample size may have hindered the ability to detect relationships between some of the study variables. In addition, the sample was of individuals accepted by the EIP services in Cambridgeshire and Peterborough only. This may have limited the generalisability of results to other areas serving different populations. For example, most patients were from a White British background (62%) and other ethnic groups were collapsed into two broad categories (White Non-British and Any Other Ethnic group). Any variations in access to EIP between subgroups were consequently missed. The study is also limited by the cross-sectional design, and therefore it is not possible to infer causality. Furthermore, the CPFTRD consists of de-identified clinical information recorded by clinicians and administrative staff. The accuracy of this information is dependent on the quality and detail of documentation.

### Research and clinical implications

This study provides important exploratory findings about sociodemographic and PtC variables associated with accessing EIP during ARMS compared to FEP in the UK. Future research with larger sample sizes across diverse catchment areas is warranted to validate these findings. Additionally, future research would benefit from investigating differences in PtC between ARMS and FEP individuals prior to entering secondary mental health services, including primary services, non-healthcare professionals, and informal help-seeking contacts with family/friends. This would provide a more complete picture of individual’s PtC and factors associated with accessing help at an earlier stage. Given the short duration of PtC observed within secondary mental health service, understanding pathways prior to accessing such services may highlight where significant delays to treatment occur and therefore where interventions to reduce such delays may be most meaningfully targeted.

Intervention studies aimed at improving access to treatment would be beneficial. It would be useful for interventions to raise awareness about early signs, the importance of early treatment, and how to access care in groups less likely to access help during ARMS. For example, potential patients and their families, and organisations working with young people, men, or individuals from ethnic minority backgrounds. Our findings, and those of previous research, suggest there are some differences in sociodemographic [24–28], PtC [11, 23], and clinical characteristics [7] between ARMS and FEP groups. Given this, the development of different approaches to promote earlier engagement in treatment is warranted for the two groups. Current evidence for early detection interventions is mixed, however strategies have often been aimed at broad groups [69, 70] and a recent systematic review identified no studies investigating public health interventions aimed at reducing delays in ARMS populations [70]. It may be that targeted interventions have more promising results, indeed public health interventions aimed at reducing delays to treatment in FEP populations appear to produce greater reduction in DUP for different groups, for example men or single individuals [70]. EIP services are situated within their local communities and are therefore well positioned to deliver such interventions with the aim to reduce delays and improve outcomes [1]. For such interventions to be feasible, it is vital that commissioning groups and policy makers ensure funding and resources are made available. In the UK, mental health services have experienced underfunding which has impacted on access and provision of care [71]. This has led to more focus on acute, rather than preventative interventions [72].

PtC depend on the accessibility of local mental health services [29]. It is therefore vital to consider whether clinical services are sensitive and responsive to the needs of the populations they serve. To do this, it would be helpful for services to work alongside their local communities to understand their needs, preferences, and potential barriers to care and develop strategies to address these [48, 69, 73]. For example, community and religious leaders are important help-seeking contacts for some individuals from ethnic minority groups [74] and would be invaluable collaborators to help services ensure they are culturally sensitive and accessible.

## Conclusion

Further research is required to replicate these preliminary findings and to investigate the effectiveness of interventions aimed at facilitating access to EIP at an earlier stage of illness to improve outcomes.

## Data Availability

CPFTRD data cannot be made publicly available.
